# Systems biology reveals reprogramming of the S-nitroso-proteome in the cortical and striatal regions of mice during aging process

**DOI:** 10.1038/s41598-020-70383-6

**Published:** 2020-08-17

**Authors:** Maryam Kartawy, Igor Khaliulin, Haitham Amal

**Affiliations:** School of Pharmacy, Faculty of Medicine, Institute for Drug Research, The Hebrew University of Jerusalem, Jerusalem, Israel

**Keywords:** Molecular neuroscience, Gene ontology, Proteome informatics, Proteomics

## Abstract

Cell aging depends on the rate of cumulative oxidative and nitrosative damage to DNA and proteins. Accumulated data indicate the involvement of protein S-nitrosylation (SNO), the nitric oxide (NO)-mediated posttranslational modification (PTM) of cysteine thiols, in different brain disorders. However, the changes and involvement of SNO in aging including the development of the organism from juvenile to adult state is still unknown. In this study, using the state-of-the-art mass spectrometry technology to identify S-nitrosylated proteins combined with large-scale computational biology, we tested the S-nitroso-proteome in juvenile and adult mice in both cortical and striatal regions. We found reprogramming of the S-nitroso-proteome in adult mice of both cortex and striatum regions. Significant biological processes and protein–protein clusters associated with synaptic and neuronal terms were enriched in adult mice. Extensive quantitative analysis revealed a large set of potentially pathological proteins that were significantly upregulated in adult mice. Our approach, combined with large scale computational biology allowed us to perform a system-level characterization and identification of the key proteins and biological processes that can serve as drug targets for aging and brain disorders in future studies.

## Introduction

Nitric oxide (NO) is produced in different organs and tissues, including the central and peripheral nervous system, and is one of the most important signaling molecules in the body^1,2^. At low concentrations, it participates in cell signaling and may have therapeutic value for brain injury^3^. However, at higher concentrations, NO may incur cell damage and death^4,5^. Thus, the chemical reactions of the target proteins containing the sulfhydryl groups of cysteine with NO cause posttranslational modifications (PTMs) leading to the formation of *S*-nitrosothiols producing *S*-nitrosylated proteins^6^. Protein S-nitrosylation (SNO) regulates the localization and activity of many key enzymes and receptors^4,7^ resulting in modulation of signal transduction pathways, synaptic plasticity, axonal elongation, protein assembly and movement of proteins to the cell membrane^4,8^. SNO can be a mediator of different kinds of brain disorder, such as Alzheimer's disease (AD)^9–11^, Parkinson’s disease (PD)^12^, Huntington’s disease (HD)^4,9^, and other neurodegenerative diseases^10,13–15^. Recently, we have also found SNO involvement in an autism spectrum disorder mouse model^16^. Abnormal protein SNO can cause protein misfolding, synaptic damage, mitochondrial fission, or apoptosis^6^. Recently, we have found differences in SNO between both sexes^18^.

Brain aging can weaken the endogenous defence systems, such as antioxidant enzyme and molecular chaperone systems, elevating NO levels in the brain. The accumulated data suggest that excessive production of NO is attributed to nitrosative stress, which exacerbates the disease-related risk factors^4^. Thus, it has been shown that the increase in oxidative/nitrosative stress is accompanied by the mitochondrial dysfunction, increase in superoxide levels, RNA oxidation, NADPH oxidase (NOX) activity, and inducible nitric oxide synthase (iNOS) expression in the hippocampal astrocytes isolated from aged and adult rats^19^. The inflammatory response and oxidative/nitrosative stress in these cells could be mediated by NFκB because its activation brings about increased expression of iNOS, resulting in elevated NO production and proinflammatory cytokine release. The MAPK pathway has also been implicated in oxidative/nitrosative stress and inflammatory response through p38 MAPK and NFκB activation^19^. Aged-related increase in SNO of the small GTPase Dexras1 has also been discovered by Zhang et al.^20^ in APP/PS1 mice, a transgenic mouse model of Alzheimer’s disease.

S-nitrosoglutathione reductase (GSNOR) expression has found to be reduced in primary senescent cells during aging in rodents and humans^17^. This was accompanied by the increased level of SNO of Dynamin-related protein 1 (Drp1) and Parkin with the downstream effect of the impaired mitophagy, pointing to mitochondrial nitrosative stress^17^. Likewise, Rizza et al. have shown that due to epigenetic events, GSNOR expression declined with age in the GSNOR-deficient mice promoting mitochondrial nitrosative stress, including excessive SNO of Drp1 and Parkin, and resulted in the impairment of mitochondrial dynamics and mitophagy^21^. Popa-Wagner et al. have also recently demonstrated the age-related imbalance in the processes of mitochondrial biogenesis and mitophagy, particularly related to the complexes III and IV of the electron transport chain, that may have a negative impact on the energy production in the cerebellum in old mice^22^. These results can be supported by the data^23^ showing that excessive SNO in aging impairs the E3 ubiquitin ligase activity of Parkin, and its ability to act as an enhancer of mitophagy^24,25^. Interestingly that excessive SNO accompanied by GSNOR deficiency may also inhibit mitophagy independently of the PINK/Parkin pathway^21^. Thus, the accumulated data indicate the increased protein SNO in the brain of aged animals and long-lived humans. However, the changes in SNO in aging and specifically from young to adult age remain unknown. In this study, we investigate SNO in aging process and compare for the first time the effects and mechanisms of protein SNO in the brain of the juvenile (6–8 week-old) and adult (3–5 month-old) mice in both cortical and striatal regions.

## Results

To test the hypothesis that age regulates NO production, which may lead to reprogramming of the S-nitroso-proteome, we mapped the S-nitroso-proteome in WT juvenile (J, 6–8 weeks-old) and adult mice (A, 3–4 months-old) using SNOTRAP-based mass-spectrometry technology^16,26^. Two regions, the cortex and striatum, were studied for their well-known role in brain disorders such as ASD, AD, HD and others^27–32^. This was followed by systems biology analysis combined with bioinformatic to gain the systems-level insight into SNO-proteins’ functionalities, and to test whether enriched processes are changed within age. Four groups were tested: J-cortex, J-striatum, A-cortex, and A-striatum. Further, large scale quantitative analysis of the overlapped SNO-proteins between the two ages was conducted in the cortex and in the striatum.

### Age leads to reprogramming of the S-nitroso-proteome in both cortex and striatum regions

**Figure 1:**
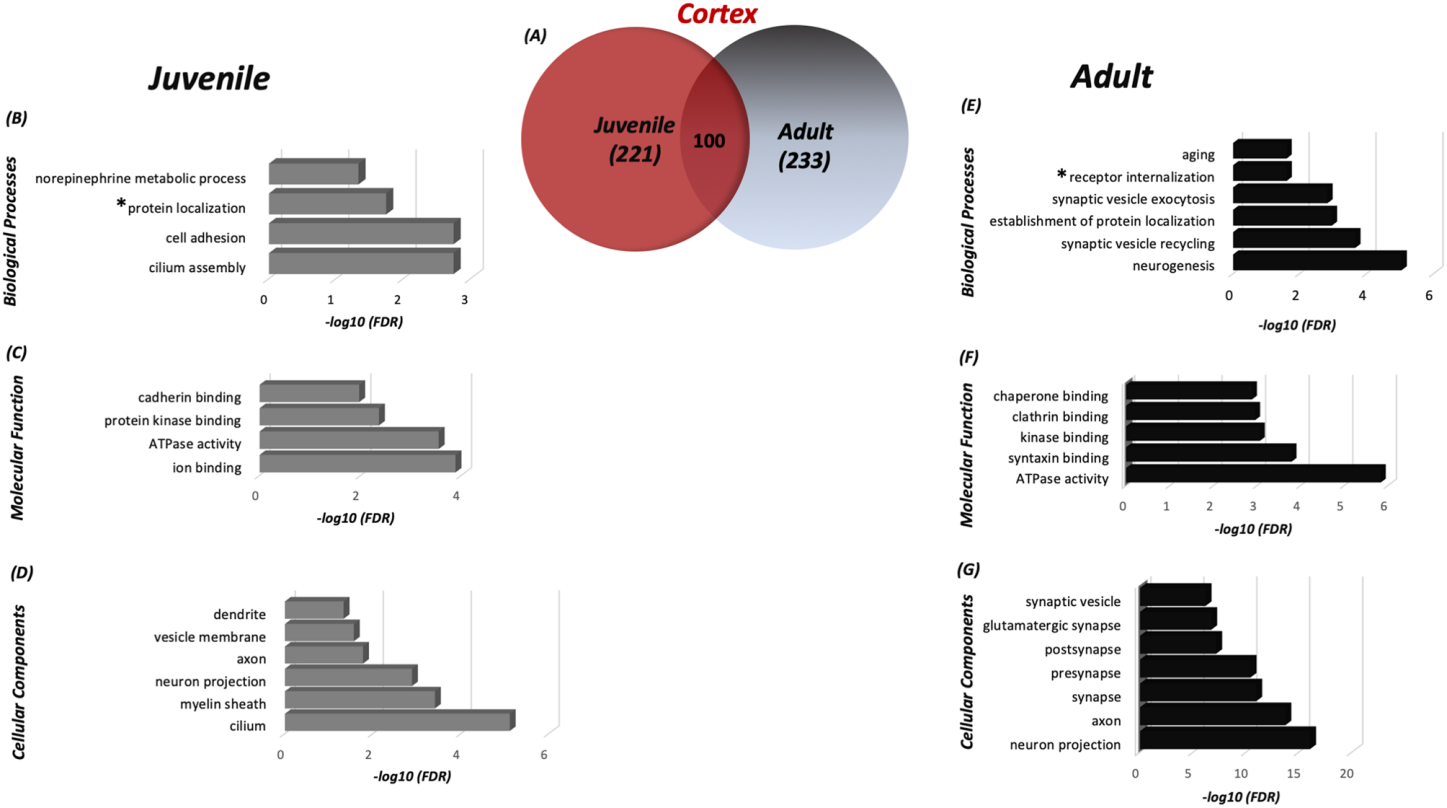
Differences in the S-nitroso-proteome between the cortex of adult and juvenile mice according to the systems biology analysis. (**a**) Venn diagram representing the SNO-proteins that were identified in the juvenile and adult cortex. Biological processes (BP) (**b**), molecular functions (MF) (**c**) and cellular components (CC) (**d**) analysis was conducted on the SNO proteins exclusive to the juvenile cortex. BP (**e**), MF (**f**), and CC (**g**) analysis was conducted on the SNO proteins exclusive to the adult cortex. *Protein localization regulation of the protein localization to the membrane; *Receptor internalization neurotransmitter receptor internalization. MetaCore from Clarivate Analytics (MetaCore version 6.34 build 69200) was used to generate this figure.

**Figure 2:**
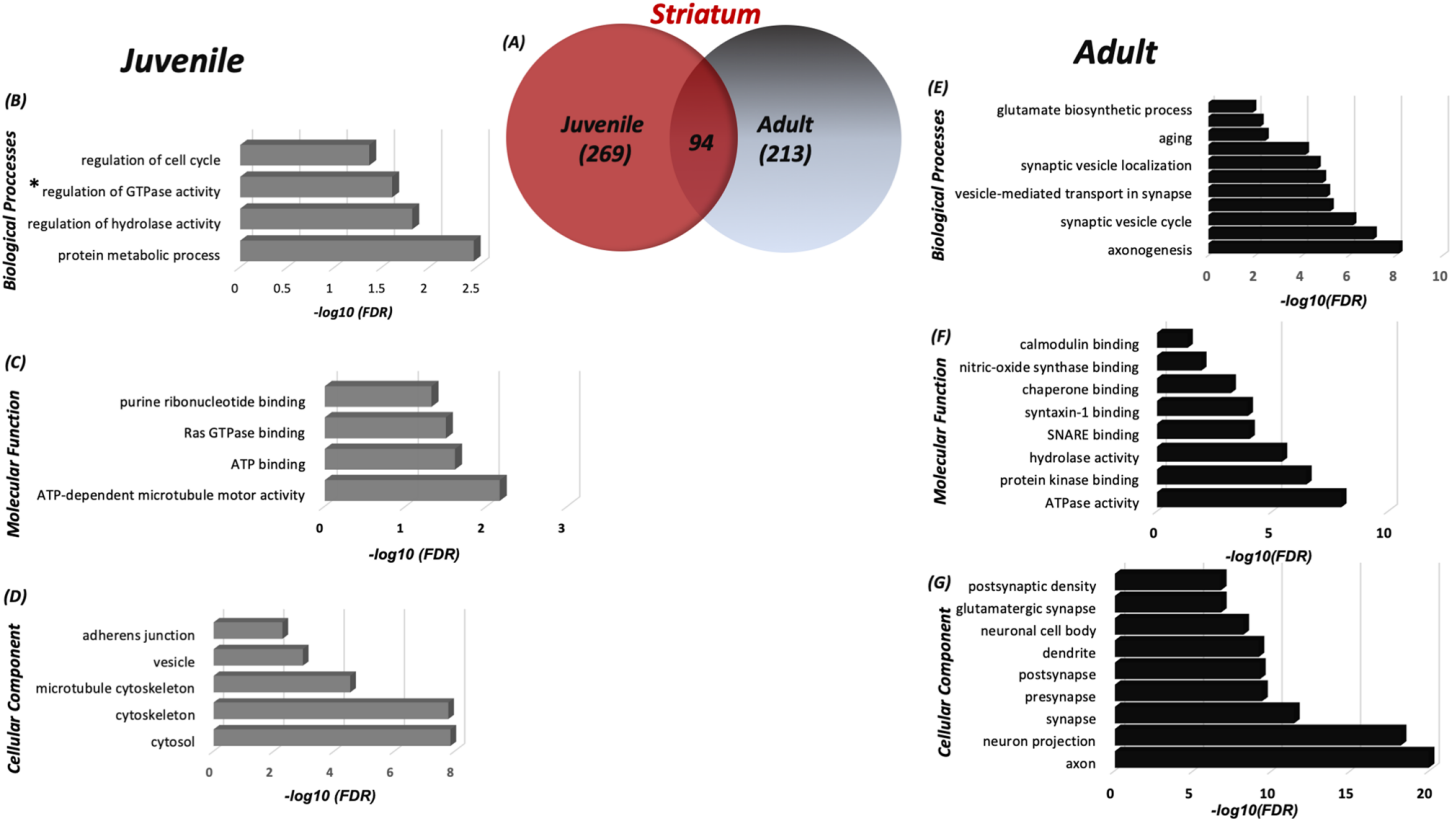
Differences in the S-nitroso-proteome between the striatum of adult and juvenile mice according to the systems biology analysis. (**a**) Venn Diagram representing the SNO-proteins that were identified in the juvenile striatum and adult striatum. BP (**b**), MF (**c**) and CC (**d**) analysis was conducted on the SNO proteins exclusive to the juvenile cortex. BP (**e**), MF (**f**), and CC (**g**) analysis was conducted on the SNO proteins exclusive to the adult striatum. *Regulation of GTPase activity—*positive regulation of GTPase activity*. MetaCore from Clarivate Analytics (MetaCore version 6.34 build 69200) was used to generate this figure.

Identification of the SNO-proteins using SNOTRAP technology revealed reprogramming of the S-nitroso-proteome in age dependent manner. Different sets of proteins were detected in juvenile and adult mice in both cortex and striatum regions. In the cortex, we identified a total of 221 proteins that were SNOed exclusively in juvenile mice, and a total of 233 proteins were SNOed exclusively in adult mice (see Fig. 1a). In the striatum, 269 proteins were SNOed in juvenile mice only, and total of 213 proteins were SNOed only in adults (see Fig. 2a). The list of the tested proteins of each group can be found in Supplementary Table S1.

### Systems biology analysis of the S-nitroso-proteome in juvenile and adult mice

To better understand the underlying biological and functional processes of the SNO-proteins, we performed a large-scale systems biology analysis by dissecting the Biological processes (BP), molecular functions (MF) and cellular components (CC). In both J-cortex and J-striatum groups, the BP analysis revealed significant enrichment of general metabolic and biological regulation processes. On the other hand, in A-cortex and A-striatum, the analysis revealed that the SNO proteins significantly enriched synaptic and neuronal-associated processes.

In juvenile cortex, BP analysis showed significant enrichment of norepinephrine metabolic processes (False discovery rate (FDR) = 0.00098), regulation of protein localization to membrane (FDR = 0.0023) etc. (see Fig. 1b), whereas, in adult cortex, BP analysis indicated enrichment of neurogenesis (FDR = 9.10E−06), synaptic vesicle exocytosis (FDR = 0.0015) etc. (see Fig. 1e). In J-cortex, the MF analysis revealed significant enrichment of ATPase activity (FDR = 0.00028) and ion binding (FDR = 0.00013) (see Fig. 1c), whilst, in A-cortex, the MF analysis showed enrichment of the biochemical activities that are associated with synaptic and neuronal processes, such as syntaxin binding (FDR = 0.00016), clatherin binding (FDR = 0.0011) and others (see Fig. 1f). Also, in J-cortex, the CC analysis demonstrated that the SNO proteins were significantly enriched in axon (FDR = 0.000253), cilium (FDR = 7.02E−06) and others (see Fig. 1d). In A-cortex, the CC analysis showed significant enrichment of glutamatergic synapse (FDR = 2.03E−07), pre-synapse (FDR = 3.89E−11) and others (see Fig. 1g).

In the striatum of juvenile mice, the BP analysis revealed enrichment of protein metabolic processes (FDR = 0.0034), regulation of cell cycle (FDR = 0.0433) etc. (see Fig. 2b). This was different compared to the striatum of adult mice, where the BP analysis revealed significant enrichment of synaptic vesicle cycle (FDR = 6.71E−07), axonogenesis (FDR = 7.3E−09) and others (see Fig. 2e). In J-striatum, at MF level, the SNO proteins were significantly enriched in ATP binding (FDR = 0.0264), Ras GTPase binding (FDR = 0.0337) and others (see Fig. 2c). In A-striatum, the MF analysis showed enrichment of SNARE binding (FDR = 9.24E−05), calmodulin binding (FDR = 0.0462), and others (see Fig. 2f). In J-striatum, the CC analysis showed significant enrichment of cytosol (FDR = 1.39E−08), cytoskeleton (FDR = 1.64E−08) and others (see Fig. 2d). Meanwhile, the CC analysis in the adult striatum displayed significant enrichment of axon (FDR = 1.08E−20), post-synaptic density (FDR = 1.81E−07) and others (see Fig. 2g).

The detailed lists of the BP, MF and CC enriched in all 4 tested groups can be found in Supplementary Table S2 (J-cortex), Table S3 (A-cortex), Table S4 (J-striatum) and Table S5 (A-striatum).

To test the shared affected signaling pathways of adult mice in the two regions, we conducted pathway analysis of the SNO proteins in the cortex and striatum. The analysis revealed significant enrichment in neuronal and synaptic pathways (see Supplementary Fig. S1). For example, “Adult neurogenesis” pathway was enriched with FDR = 9.12E−06. “Synaptic vesicle fusion and recycling” pathway was also significantly enriched with FDR = 8.54E−04. The detailed cell trafficking of both signaling pathways is described in Supplementary Figs. S2 and S3, respectively.

### Proteins classification analysis in the cortex and striatum of both ages

Proteins Classification enrichment analysis was conducted to examine the functions of the SNO-proteins in both ages. We identified diverse protein families in both juvenile and adult mice, such as hydrolases, receptors, transferases, ligases, immunity proteins, etc**.**

We found a higher number of SNOed kinases (e.g. AAK1, NADK and others) and phosphatases (e.g. SYNJ1, DNML1, PTPRA and others) in adult mice compared to juvenile mice in the cortex (see Supplementary Fig. S4), which may lead to alteration of critical signaling pathways in adults. Phosphatases, such as SYNJ1 and DNML1, play an essential role in synaptic vesicle recycling, regulation of endocytosis and regulation of neurotransmitter levels^33^. SNOed kinases also play a critical role in brain functioning. For example, NAD kinase acts as a mediator of calcium homeostasis^34^, AP-2 associated kinase 1 (AAK1) has a vital role in clatherin-mediated endocytosis^35^ and so forth.

In Striatum, no difference in the SNOed phosphatases of both ages was found. Two essential kinases SNOed in adult striatum, the PRKCA and PIP5K1, are functionally related to the synaptic vesicle endocytosis^36,37^ (see Supplementary Fig. S4).

### Protein–protein interaction network and clustering analysis of the S-nitroso-proteome

To test whether clusters of SNO-proteins orchestrate in critical biological processes, we conducted functional and physical interactions network analysis (for all networks and clusters, see Supplementary Figs. S5, S6, respectively).

Physical interaction analysis revealed a total of 245 and 273 interactions in the juvenile cortex and juvenile striatum, respectively. BP analysis of the clusters showed that they are involved in multiple general cellular and metabolic processes, such as metabolism, regulation and developmental processes, including regulation of protein localization to membrane, regulation of cell cycle and others. A total of 548 and 635 interactions found in the adult cortex and adult striatum, respectively, appeared to be involved in the synaptic and neuronal processes, such as vesicle-mediated transport in the synapse, neurotransmitter receptor internalization and others.

**Figure 3:**
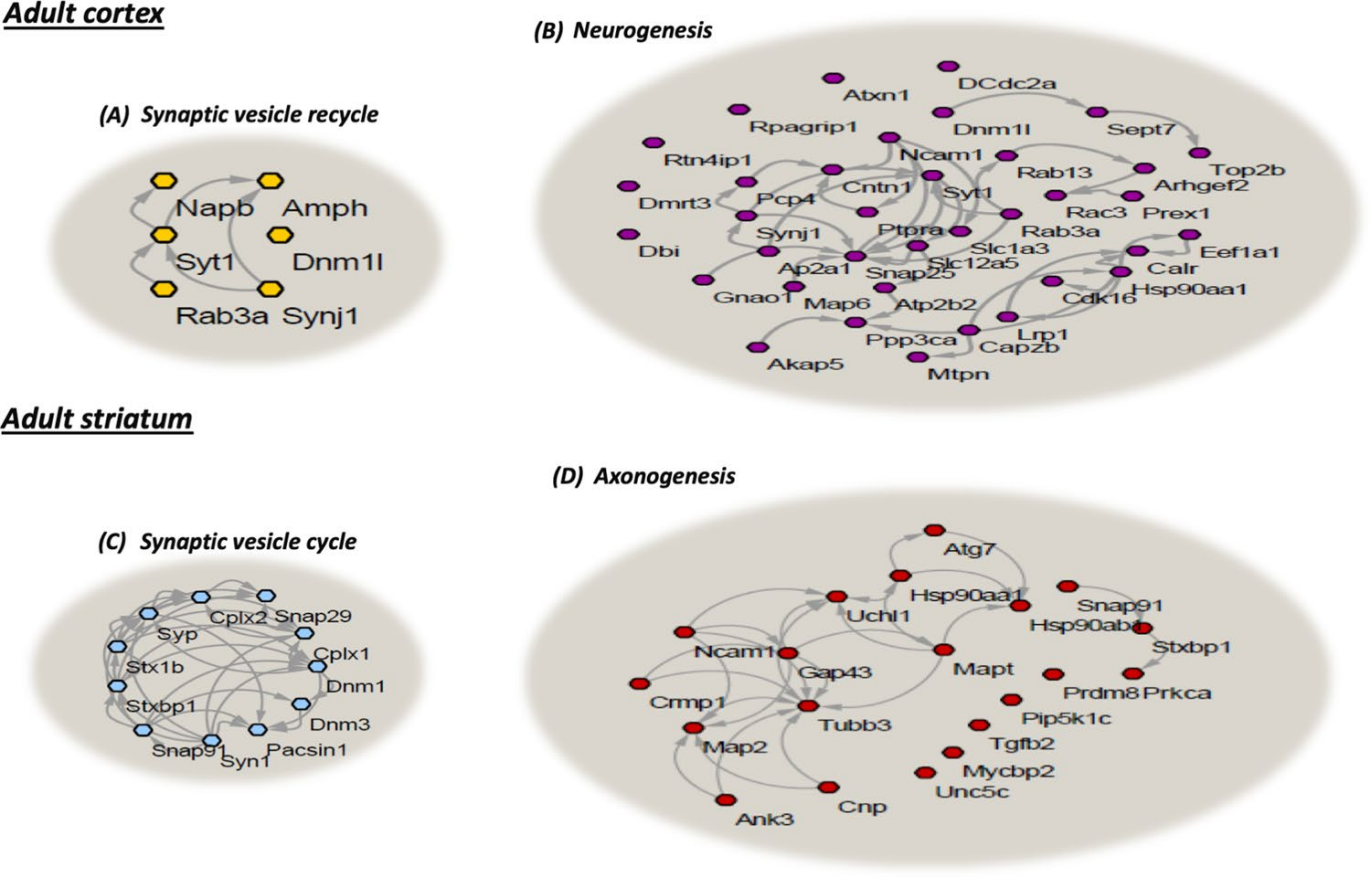
Clustering analysis of the cortical and striatal SNO-proteins. The synaptic vesicle recycling in the adult cortex (**a**; n = 6, FDR = 0.00022) and neurogenesis (**b**; n = 42, FDR = 9.10E−06) clusters were enriched. The synaptic vesicle cycle in the adult striatum (**c**; n = 11, FDR = 6.71E−07) and axonogenesis (**d**; n = 20, FDR = 7.30E−09) clusters were enriched. STRING, version 10.0 and Cytoscape software version 3.3.0 were used to generate this figure.

To get further functional insight into the data, we applied functional clustering analysis to the SNOed proteins found exclusively in the cortex and striatum of the adult mice. We used the gene ontology (GO) classification system in order to classify the proteins into different clusters based on their enriched biological processes (see Fig. 3). Several of the identified cortical and striatal SNOed protein clusters formed four distinct functional clusters. These clusters showed to be involved in the neuronal and synaptic processes. In the adult cortex, the yellow nodes correspond to the SNOed proteins involved in synaptic vesicle recycling. The cluster included AMPH, SYT1, NAPB and others (see Fig. 3a). The purple nodes correspond to the SNOed proteins involved in the neurogenesis. These were DNM1L, SYNJ1, SYT1, NCAM1, SNAP25, LRP1 and other proteins (see Fig. 3b). In the adult striatum, the blue nodes correspond to the SNOed proteins involved in the synaptic vesicle cycle. They included SYN1, DNM3, STX1B, SYP, CPIX1 and others (see Fig. 3c). The red nodes correspond to the SNOed proteins, including SNAP91, STXBP1, MAPT, CNP, ATG7 and others. These proteins were found to be involved in the axonogenesis (see Fig. 3d).

### Quantitative analysis of the S-nitroso-proteome

To promote a deeper and further understanding of the changes (the reprogramming) in S-nitroso-proteome, we performed a large-scale quantitative analysis. We studied the shared proteins that were SNOed in adult and in the juvenile cortex and striatum.

Volcano plot analysis was conducted to visualize and identify the significant changes that occur among the SNOed proteins at the two different ages. The fold change (log2(FC)) was plotted against the level of statistical significance (− log10(P value)) on X and Y axes, respectively. The fold change was calculated as the difference of the protein’s abundance in both ages divided to the protein’s abundance at the juvenile state. Statistical significance thresholds of FC > 1.3 and P < 0.05 indicate significantly upregulated proteins, and of FC <  − 1.3 and P < 0.05 indicate significantly downregulated proteins.

**Figure 4:**
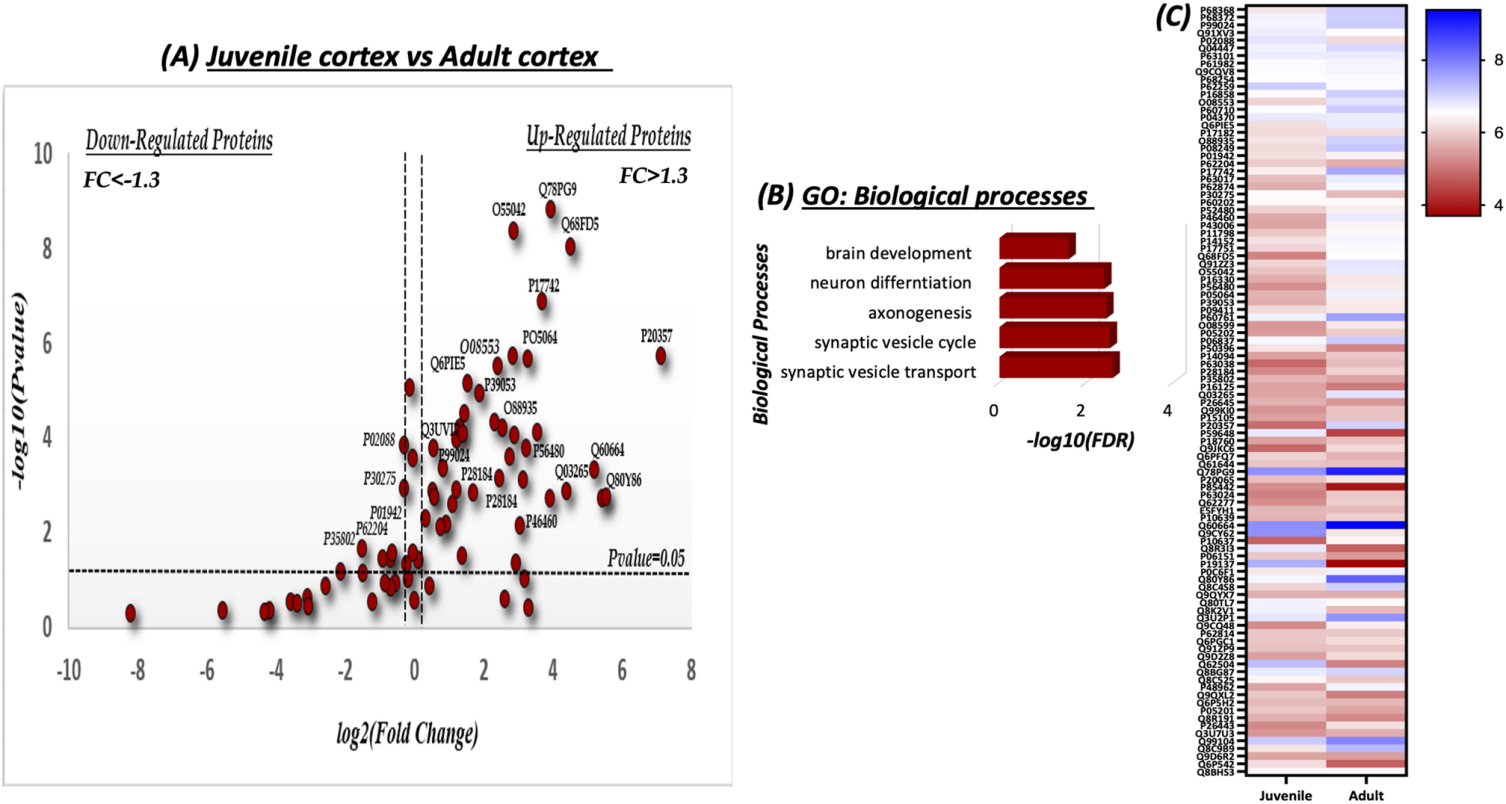
Quantitative analysis of the shared SNO-proteins in the juvenile (J) and adult (A) cortex. (**a**) Volcano Plot analysis was conducted on the shared SNO proteins in the J cortex *vs.* A cortex. The fold change (FC) was calculated as the difference of the relative abundance between A and J divided to J [FC = $\frac{{{\text{Relative}}{\mkern 1mu} {\text{abundnce}}\left( {\text{A}} \right) - {\text{Relative}}{\mkern 1mu} {\text{abundance}}({\text{J}})}}{{{\text{relative}}{\mkern 1mu} {\text{abundance}}({\text{J}})}}$] and normalized using log2. The horizontal line represents a significance level of P value= 0.05. The vertical lines represent the threshold of the FC = 1.3. The upregulated proteins are those that appear on the right side of the plot. These proteins meet the statistical significance criteria of both P < 0.05 and a FC > 1.3. (**b**) Biological processes of the significant up-regulated SNO-proteins. (**c**) Heat map analysis representing the differential relative abundance of the shared SNO-proteins in the J and A cortex. The abundance scale was normalized by – log10. Prism-GraphPad 8 was used to generate this figure.

In the cortex, 100 proteins were identified as shared proteins between the two ages. Among them, 34 proteins appeared to be significantly upregulated in adult cortex (see Fig. 4a). Strikingly, GO enrichment analysis of the significantly upregulated proteins revealed the involvement of these proteins in the neurodevelopmental and synaptic processes, such as synaptic vesicle cycle (SYN1, DNM1, STXBP1), brain development (SLCLA2, CNP, MT3, DPYSI2) and other processes (see Fig. 4b). Heat map analysis was conducted to visualize the quantitative differences of the relative abundance of the shared proteins between the juvenile and adult cortex (see Fig. 4c).

**Figure 5:**
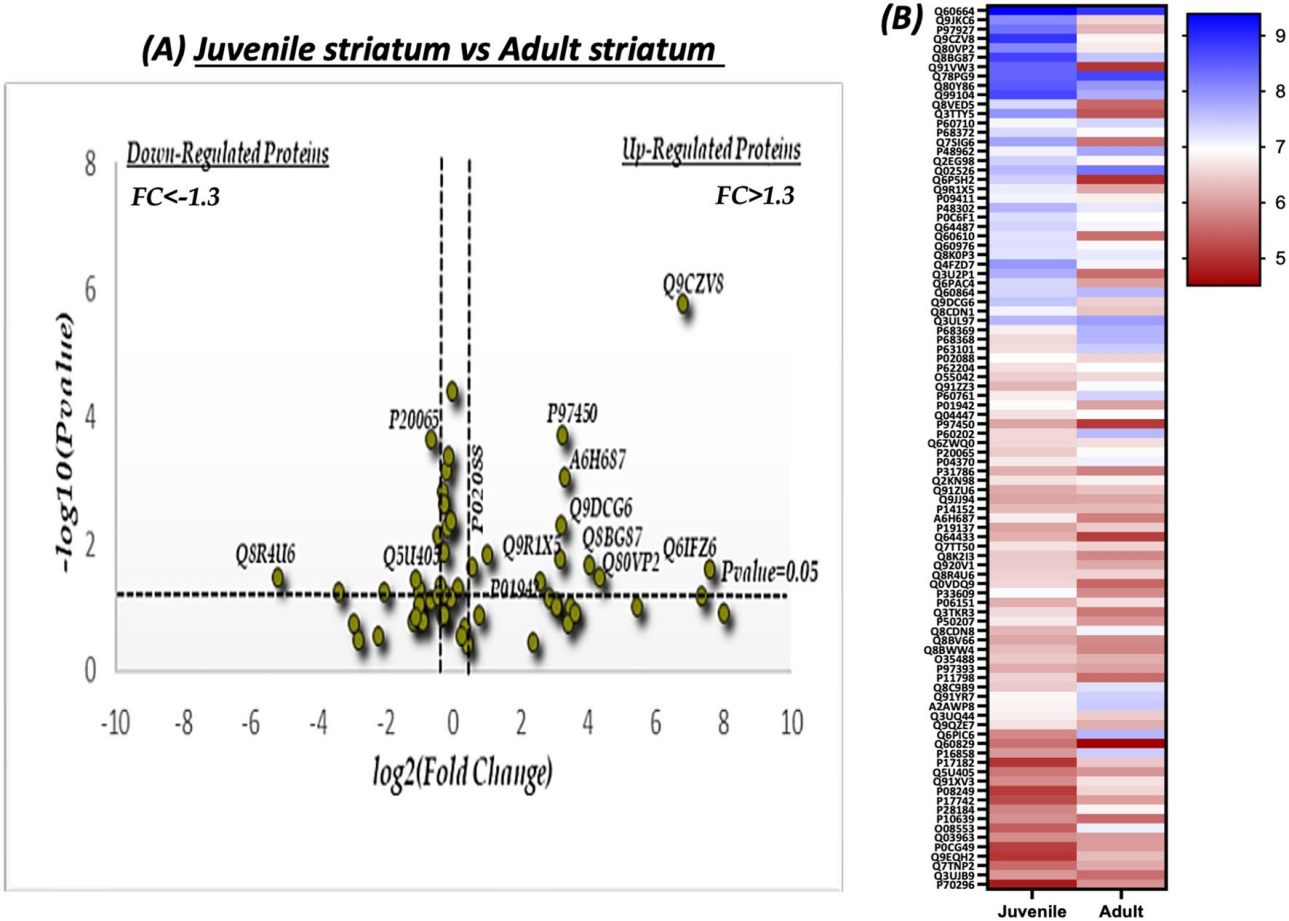
Quantitative analysis of the shared SNO-proteins in the adult (A) and juvenile (J) striatum. (**a**) Volcano Plot analysis was conducted on the shared SNO Proteins in the J *vs.* A striatum. The fold change (FC) was calculated as the difference of the relative abundance between A and J divided to J [FC = $\frac{{{\text{Relative}}{\mkern 1mu} {\text{abundnce}}\left( {\text{A}} \right) - {\text{Relative}}{\mkern 1mu} {\text{abundance}}({\text{J}})}}{{{\text{relative}}{\mkern 1mu} {\text{abundance}}({\text{J}})}}$] and normalized using log2. The horizontal line represents a significance level of P = 0.05. The vertical lines represent the threshold of the fold change (FC) = 1.3. The upregulated proteins are those that appear on the right side of the plot. These proteins meet the statistical significance criteria of both P < 0.05 and a FC > 1.3. (**b**) Heat map analysis representing the differential relative abundance of the shared SNO-proteins in the juvenile and adult striatum. The abundance scale was normalized by − log10. Prism-GraphPad 8 was used to generate this figure.

In the striatum, 94 proteins were identified as shared proteins between the two ages. 11 proteins out of the 94 were significantly upregulated in the adult striatum (see Fig. 5a). Due to the small number of upregulated proteins; GO analysis showed no significant difference in the BP analysis compared to the juvenile cortex. Heat map analysis was conducted to visualize the quantitative differences of the relative abundance of the shared proteins between the juvenile and adult striatum (see Fig. 5b).

## Discussion

In our work, we employed the novel MS method SNOTRAP, to identify the S-nitroso-proteome, combined with systems biology analysis, to test the hypothesis that aging leads to changes of the NO biochemistry and to reprogramming of the S-nitroso-proteome in the cortex and striatum of adult mice. Here we would like to emphasize, that although a popular understanding of the term “aging” can be described by the definition: “Age-progressive decline in intrinsic physiological function, leading to an increase in age-specific mortality rate and a decrease in age-specific reproductive rate”^38^, in our work, we use this term with a broader meaning of the “age-related changes”, which does not necessarily mean becoming old. This approach is consistent with the Developmental Aging Theory, proposed by Dilman in 1971^39^, suggesting that “aging is part of the same molecular mechanism that promotes tissue development and embryonic maturation during development, continuing throughout adult life”^40,41^.

Our study combined SNOTRAP-based MS technology that enables global identification of SNO proteins^16,26^ with system biology analysis and bioinformatics. This allowed us to identify the key processes and pathways that are enriched among the proteins undergoing S-nitrosylation in the brain^42^. We tested the cortex and striatum of the juvenile mice (6–8 weeks) and adult mice (3–5 months). We conducted BP, pathways and clusters analysis of both ages and found that aging regulates NO and leads to reprogramming of the S-nitroso-proteome, resulting in the changes in biological and functional processes in the adult mice compared to the juvenile mice.

S-nitrosylation is the NO-mediated PTM that regulates protein function leading to modulation of the signal transduction pathways^4,8,16,43^. Growing body of evidence has showed that under the pathophysiological conditions, aberrant S-nitrosylation plays a fundamental role in the pathogenesis of neurodegenerative disorders, including ASD^16^, AD^4,8,11,16^, PD and HD^4,8,9,12,16^. Thereby, we suggest that NO and S-nitrosylation play a major role in aging, which is one of the risk factors of multiple neurodegenerative diseases.

The analysis of the SNO proteins exclusive for adult cortex and adult striatum revealed enrichment of the neuronal and synaptic associated processes that previously have been reported to be affected in brain disorders. This points to the possibility that S-nitrosylation has a key role in neurodegenerative disorders. For example, in both adult cortex and adult striatum, the BP analysis showed enrichment of the biological events associated with synaptic and vesicle processes, such as synaptic vesicle recycling, vesicle-mediated transport in the synapse, neurotransmitter receptor internalization and synaptic vesicle localization (see Figs. 1e, 2e). Our data is consistent with the previous investigations that revealed the involvement of these processes in the progression of neurological and neurodegenerative disorders, including ASD, AD, PD, HD, schizophrenia and bipolar disorder^44–47^.

Further, pathway analysis of the SNO-proteins in the cortex and striatum of the adult mice revealed enrichment of neuronal and synaptic pathways that had been proposed previously as major pathways of neurodegenerative disorders (see Supplementary Fig. S1). For example, this included “adult neurogenesis in the subventricular zone” and “synaptic vesicle fusion and recycling in nerve terminals” (see Supplementary Fig. S2, S3). One of the major processes that was found to be significant in the adult mice was “neurogenesis”. Perturbations in neurogenesis have been suggested as an early and common hallmark in neurological disorders including AD, PD, HD, epilepsy and schizophrenia^48–53^. NCAM1 is one of the proteins that appeared to be S-nitrosylated in the adult cortex and adult striatum and found to be involved in neurogenesis. It is neuronal cell adhesion molecule that plays a key role in synaptic plasticity, neuronal migration and axonal\dendritic growth. Recent studies suggest that these functions may be altered in the brain disorders, thus implicating NCAM1 in the pathogenesis of brain disorders including AD, PD, bipolar depression and schizophrenia^54^. Due to the fact that S-nitrosylation can modulate protein function, we suggest that SNO-NCAM1 might provide a potential mechanism of the pathogenesis of neurological disorders.

Synaptic vesicle fusion and recycling process, which were enriched in the adult groups, represent a pivotal mechanism for the synaptic transmission and neuronal function^33,55,56^. The critical proteins, which mediate the synaptic transmission were detected to be S-nitrosylated in adult cortex and the adult striatum. These were SNARE proteins (SYB, SNAP25), SNARE-associated proteins (CPLX1, SYN1, MUNC18) and endocytic protein DNM1^33,55,57,58^. Modulation by NO or uncontrolled S-nitrosylation mechanism may induce functional changes that affect synaptic transmission and might contribute to the pathogenesis of several neurodegenerative disorders^59^. Various lines of observations showed that altered expression of the synaptic proteins is linked to neurological and neurodegenerative disorders. Thus, SYB2 alteration has been identified in AD and HD^60,61^. Deletion of SNAP25 results in ADHD phenotype^62–64^. Moreover, reduced expression of SNAP25 was observed in patients with schizophrenia^65^. Changes in MUNC18 have been linked to epileptic disorders^66,67^, whilst changes in SYT1 were seen in individuals with severe cognitive and motor deficits^68^.

CPLX1 was one of the proteins S-nitrosylated in both adult cortex and adult striatum. It is a hydrophilic protein that regulates rapid neurotransmitter release by its binding to the SNARE complex with high affinity^55,69,70^. This protein functionally co-operates with SYT1 that functions as a Ca^2+^ sensor for evoked synchronous neurotransmitter release^55,71^. Recent study identified CPLX1 as a specific target for NO. At high levels of NO, CPLX1 undergoes S-nitrosylation that induces functional and synaptic localization changes and that may alter its interaction with the SNARE complex, thus leading to neurotransmission modulation^72^. Changes in CPLX have been documented in several neurodegenerative and psychiatric disorders, including HD, AD, schizophrenia and bipolar disorder^73,74^. Collectively, we suggest that SNO-CPLX1 might provide an additional mechanism for neurological disorders.

DNM1 is a large GTPase that plays an essential role in endocytosis due to the vesicle membrane fission, which can be induced by DNM1^55,75,76^. Altered expression of DNM1 has been detected in AD, epilepsy and schizophrenia^77–82^. Emerging evidence suggests that S-nitrosylation of DNM1 at Cys-607 increases the GTPase activity followed by activation of endocytosis^59,83^.

Using proteins classification enrichment analysis, we identified a higher number of SNOed phosphatases and kinases in the cortex of the adult mice (see Supplementary Fig. S4). S-nitrosylation of phosphatases and kinases can affect a wide range of signal transduction cascades. In some cases, S-nitrosylation induces an inhibitory effect on protein phosphatases (PP) and protein kinases (PK) since it suppresses the kinase and phosphatase activity^43^. Subsequently, under the pathological conditions, aberrant regulation mechanisms on PP or PK that might alter their activity or modulate their interaction with the substrates may contribute to the progression of several neurological disorders^84–87^.

In summary, our findings show that age leads to reprogramming of the S-nitroso-proteome in the adult cortex and adult striatum. The analysis reveals that age leads to S-nitrosylation of the synaptic and neuron-associated proteins that regulate the key processes and pathways known to be involved in neurological disorders. We suggest that S-nitrosylation of these proteins might contribute to the pathogenesis of several brain disorders. Our MS approach, combined with large scale computational biology allowed us to perform a system-level characterization and identification of the key proteins and biological processes that can serve as drug targets for aging and brain disorders in the future studies.

## Material and methods

### Materials and reagents

Vivapsin 10 kDa molecular weight cut off (MWCO) filters were procured from Satorious AG (Germany). For High-Performance Liquid Chromatography (HPLC) and Liquid Chromatography–Mass Spectrometry (LC–MS), HPLC grade solvents were used. Biotin-PEG3-propionic acid was obtained from Chem Pep Inc (Florida, USA). For Mass Spectrometry (MS), protease inhibitor cocktail, acetonitrile (ACN) and distilled water were purchased from Sigma-Aldrich (St. Louis, MO). Sequencing-grade modified trypsin was provided by Promega (Wisconsin, USA). SNOTRAP-biotin synthesis and Nuclear magnetic resonance (NMR) analysis were performed as described previously^26^. All methods were carried out in accordance with the Hebrew University guidelines and regulations. The animal’s data we generated previously were taken from the Pride Software as mentioned below. The animals’ experiments were done in accordance with the Institutional Animal Care and Use Committee and the Association for Assessment and Accreditation of Laboratory Animal Care International.

### Brain tissue sample preparation for mass spectrometry (MS)

All samples were prepared at room temperature in the dark.

Brain tissues were isolated from 6–8-week (Juvenile) to 3–4-month-old (adults) WT mice following decapitation during the day time. The brain samples were immediately transferred into liquid nitrogen for storage at − 80 °C for further analysis. For each biological replicate, 3–4 cortex tissue samples from 3 to 4 mice and 4 striatal tissue samples from 4 mice were pooled. Further, tissues were homogenized on ice in freshly prepared lysis buffer: 250 mM HEPES–NaOH, 0.1 mM neocuproine, 1 mM EDTA, 1% NP-40, 20 mM IAM, 1% protease inhibitors cocktail, pH 7.7. The homogenates were centrifuged (12,000–13,000×*g* for 10 min at 4 °C), the supernatant was collected and protein concentration was estimated by Bradford assay (Bio-Rad, California USA, Cat. No. 500-0006). Next, in the presence of 2.5% SDS, samples were alkylated with 30 mM IAM in the dark at 37 °C. After alkylation, samples were washed twice with 3 times volume of 8 M Urea (in 50 mM HEPES, pH 7.7) and once with 50 mM HEPES (pH 7.7) by centrifugation at 5,000×*g* for 30 min at 4 °C with 10 K MWCO spin filters pre-rinsed once with water (Satorious AG, Germany, Cat. No. VS15T01). After the centrifugation, SNOTRAP labeling stock solutions (in 50% ACN) were added to all samples to reach a final concentration of 1.25 mM. This was performed with the purpose of converting SNO to stable disulfide-iminophosphorane. Further, at 25 °C, all samples were incubated for 1.5 h in SNOTRAP solution. After the SNOTRAP labeling, reagents were removed by three consecutive washing with 50 mM HEPES (pH 7.7) buffer with 10 K filters. After ultrafiltration, each sample was incubated with 200 μl pre-rinsed Streptavidin agarose beads (Pierce, Cat. No. 20349) for 1 h at room temperature with gentle shaking. The beads were washed with washing buffer (50 mM HEPES, 150 mM NaCl, 0.1% SDS, pH 7.7) three times and then with washing buffer (50 mM HEPES, pH 7.7) three times. After washing, proteins were eluted (with 10 mM TCEP in 50 mM HEPES, pH 7.7) and then alkylated with 10 mM IAM. Protein samples were then trypsinized (Promega, Wisconsin, USA, Cat. No. V5111) at 37 °C for 4 h and then desalted with C18 StageTips as described previously^88^.

### Analysis flowchart of mass spectrometry

The digested peptides were analysed using 6550 Nano-HPLC-Chip/MS system of Agilent, coupled with a micro-autosampler, pumps of a capillary and nanoflow, and the Chip-Cube connected to the LC modules and the MS instrument. H_2_O with 0.1% FA was used as a mobile phase A and ACN with 0.1% FA was used as a mobile phase B. Polaris-HR-Chip-3C18 HPLC-Chip (Agilent Technologies, Cat. No. G4240-62030) separated the peptides. It consisted of a 360 nl enrichment column, a 75 μm × 150 mm analytical column and a 3 μm stationary phase. We loaded the peptides onto the enrichment column. The gradient was set for 55 min, starting from 3% B at 300 nl/min, increased to 30% B and kept from the 2nd to 35th min, then increased to 60% B at the 40th min, to 90% B at the 45th min and then kept stable for 5 min followed by a 5 min after-run at 3% B. We acquired the positive-ion MS spectra using 1,700 Da extended dynamic range mode: ESI capillary voltage was set on 1,960 V; fragmentor on 360 V; Octopole RF peak on 750 V; drying gas on 13 L/min; drying temperature on 225 °C. The data were acquired at the rate of 6 MS spectra/second and 3 MS/MS spectra/second in the range of m/z 300 to 1,700 for MS and 50 to 1,700 for MS/MS. The Max number of precursors per cycle was set at 20, setting the threshold at 5,000 ions in a precursor abundance-based scan speed in peptide isotope model with plus 2, plus 3 and above charge-state preference, and with active exclusion after 1 spectrum and released after 0.15 min. We set the fragmentation energy at a slope of 3.1 V/100 Da, including a 1.0 offset for doubly charged precursors, 3.6 V/100 Da with a − 4.8 offset for triply and also multiply-charged precursors. We used Agilent MassHunter Workstation software for the data acquisition. Two biological replicates with three technical replicates of each biological replicate were run. The mass accuracy was preserved using ion *m/z* 1,221.9906 as an internal reference. Detailed information on this is found in our previous work found in Ref.^16^.

### Processing of the mass spectrometry data

For peak the list generation, database searching, and false discovery rate (FDR) estimation, we used Agilent Spectrum Mill MS proteomics Workbench B.05. We used the following parameters for data extractions: precursor MH + 300–8,000 Da, scan time range from 0 to 200 min, cysteine carbamidomethylation for fixed modification, sequence tag length of > 1, default for precursor charge, true for find 12C precursor, merge scans with the same precursor at ± 30 s and ± 0.05 m/z, and MS noise threshold of 100 counts. MS/MS spectra were searched against the mouse SwissProt protein database with ± 20 ppm precursor ion tolerance and ± 50 ppm fragment ion tolerance. We included different modifications of methionine oxidation, deamidation of asparagine, and fixed modification of cysteine carbamidomethylation. The generated FDR was set to 1.2% for both peptide and protein identification. The MS proteomics data, which we have generated previously, were taken from ProteomeXchange Consortium (https://proteomecentral.proteomexchange.org) via the PRIDE partner repository with the dataset identifier < PXD006907 > and used in this study.

### Statistics and systems biology analysis

For the systems biology analysis of the Cellular Compartments (CC), Biological Processes (BP) and KEGG pathways, we uploaded the lists of all SNO-proteins into MetaCore from Clarivate Analytics (MetaCore version 6.34 build 69200). The Benjamini–Hochberg correction^89^ was used to calculate the P value and generate FDR, and terms with FDR values below 0.05 were accepted. The search tool for the interacting proteins (STRING, version 10.0) was used to analyze the protein–protein interaction of SNO-proteins (https://string-db.org)^90^. High reliability interactions (score > 0.4) from the neighborhood, gene fusion, co-occurrence, co-expression, experiments, databases and textmining lists were used. Cytoscape software (version 3.3.0) was used for visualization of the protein–protein interaction. MetaCore from Clarivate Analytics (MetaCore version 6.34 build 69,200) was used for the networks generation after submitting the lists of SNO-proteins. For this, we also used Benjamini–Hochberg correction^89^ to calculate the P value and generate FDR. The processes/terms with the FDR values of below 0.05 were included. PRISM graphpad 8 was used to generate heatmaps and Volcano plots.

## Supplementary information

Supplementary Legends

Supplementary Figure S1.

Supplementary Figure S2.

Supplementary Figure S3.

Supplementary Figure S4.

Supplementary Figure S5.

Supplementary Figure S6.

Supplementary Table S1.

Supplementary Table S2.

Supplementary Table S3.

Supplementary Table S4.

Supplementary Table S5.
