# Supplementary material for: Systems biology reveals reprogramming of the S-nitroso-proteome in the cortical and striatal regions of mice during aging process
Source: Sci Rep. 2020 Aug 17;10:13913. doi: 10.1038/s41598-020-70383-6 (PMC7431412; doi:10.1038/s41598-020-70383-6)
Supplement: Supplementary file 1 — Supplementary Legends [file 41598_2020_70383_MOESM1_ESM.pdf]

Supplementary Information

Systems biology reveals reprogramming of the S-nitroso-proteome in the cortical and striatal regions of aging mice

Maryam Kartawy[1], Igor Khaliulin[1], and and Haitham Amal[1,#]

[1] Institute for Drug Research, School of Pharmacy, Faculty of Medicine, The Hebrew University of Jerusalem, Jerusalem, Israel

# Corresponding Author: Dr. Haitham Amal, Email: Haitham.amal@mail.huji.ac.il

Supplementary legends:

Supplementary Figure S1: Pathways analysis of the SNO proteins exclusive to the adult cortex and adult striatum. MetaCore from Clarivate Analytics (MetaCore version 6.34 build 69200) was used to generate this figure.

Supplementary Figure S2: Enriched “Adult neurogenesis pathway in the subventricular zone” pathway.
Green circles are the SNO-proteins in the adult cortex. Red circles are the SNO-proteins in the adult striatum. Blue circles are the SNO-proteins in both regions. MetaCore from Clarivate Analytics (MetaCore version 6.34 build 69200) was used to generate this figure.

Supplementary Figure S3: Enriched “Synaptic vesicle fusion and recycling pathway in nerve terminals” pathway.
Green circles are the SNO-proteins in the adult cortex. Red circles are the SNO-proteins in the adult striatum. Blue circles are the SNO-proteins in both adult cortex and adult striatum. MetaCore from Clarivate Analytics (MetaCore version 6.34 build 69200) was used to generate this figure.

Supplementary Figure S4: Proteins classification enrichment analysis by PANTHER, Version 15.0. The analysis shows the SNOed phosphatases and kinases in the juvenile cortex (**a**), adult cortex (**b**), juvenile striatum (**c**) and adult striatum (**d**).

Supplementary Figure S5: Protein-Protein interaction network analysis of the SNO-proteins in the cortex and striatum.
Juvenile cortex (**a**); adult cortex (**b**); juvenile striatum (**c**); and adult striatum (**d**). STRING, version 10.0 and Cytoscape software version 3.3.0 were used to generate this figure.

Supplementary Figure S6: Clustering analysis of the SNO-proteins exclusive to the adult cortex (**a**) and adult striatum (**b**). STRING, version 10.0 was used to generate this figure.

<u>Supplementary Tables are uploaded as Excel files:</u>

Table 1: IDs of SNO-proteins in the different groups.
Table 2: Systems biology analysis of the juvenile cortex.
Table 3: Systems biology analysis of the adult cortex.
Table 4: Systems biology analysis of the juvenile striatum.
Table 5: Systems biology analysis of the adult striatum.
