# Supplementary figures and images for: Systems biology reveals reprogramming of the S-nitroso-proteome in the cortical and striatal regions of mice during aging process
Source: Sci Rep. 2020 Aug 17;10:13913. doi: 10.1038/s41598-020-70383-6 (PMC7431412; doi:10.1038/s41598-020-70383-6)

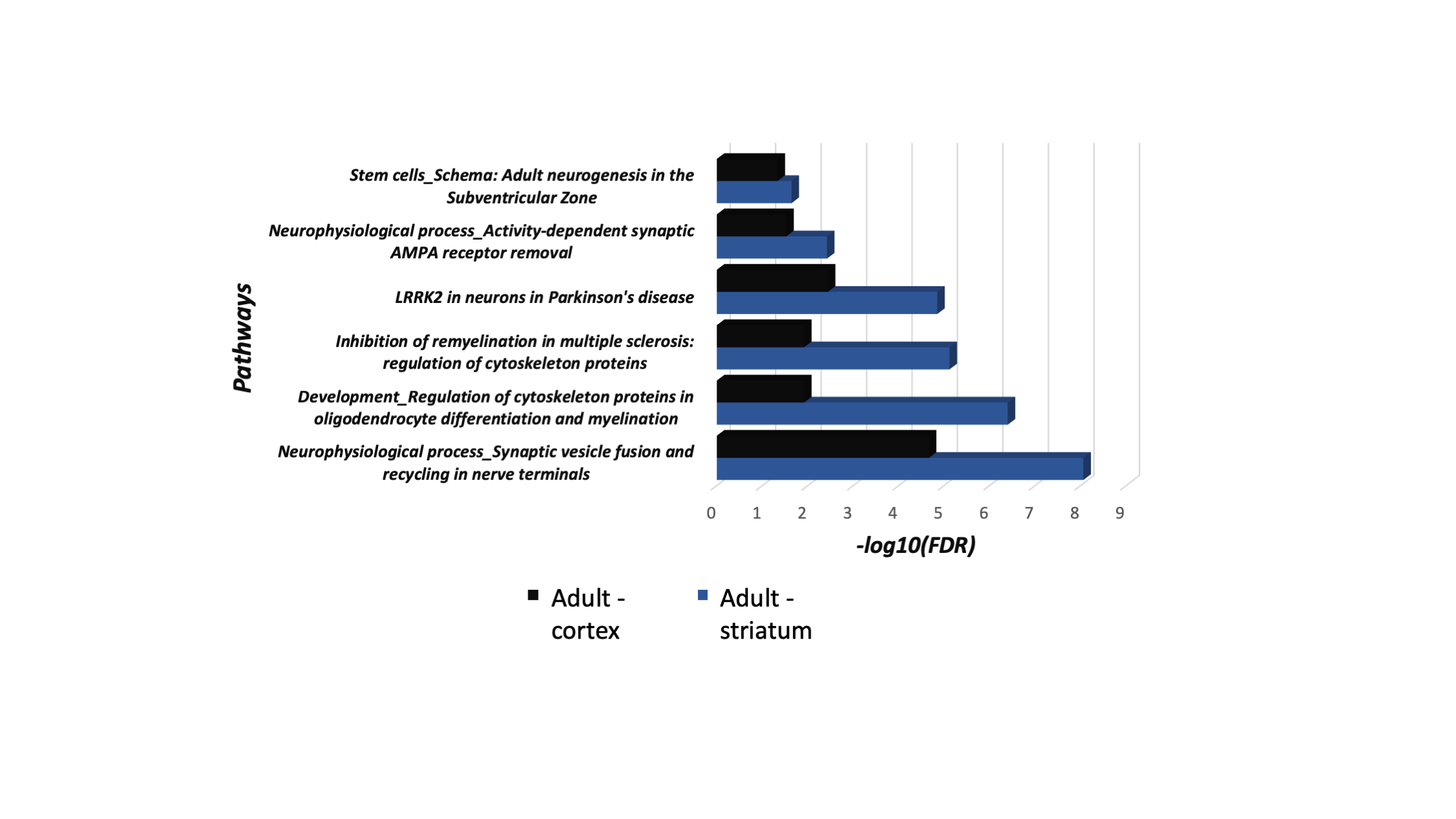

Supplement: Supplementary file 2 — Supplementary Figure S1. [file 41598_2020_70383_MOESM2_ESM.tiff]

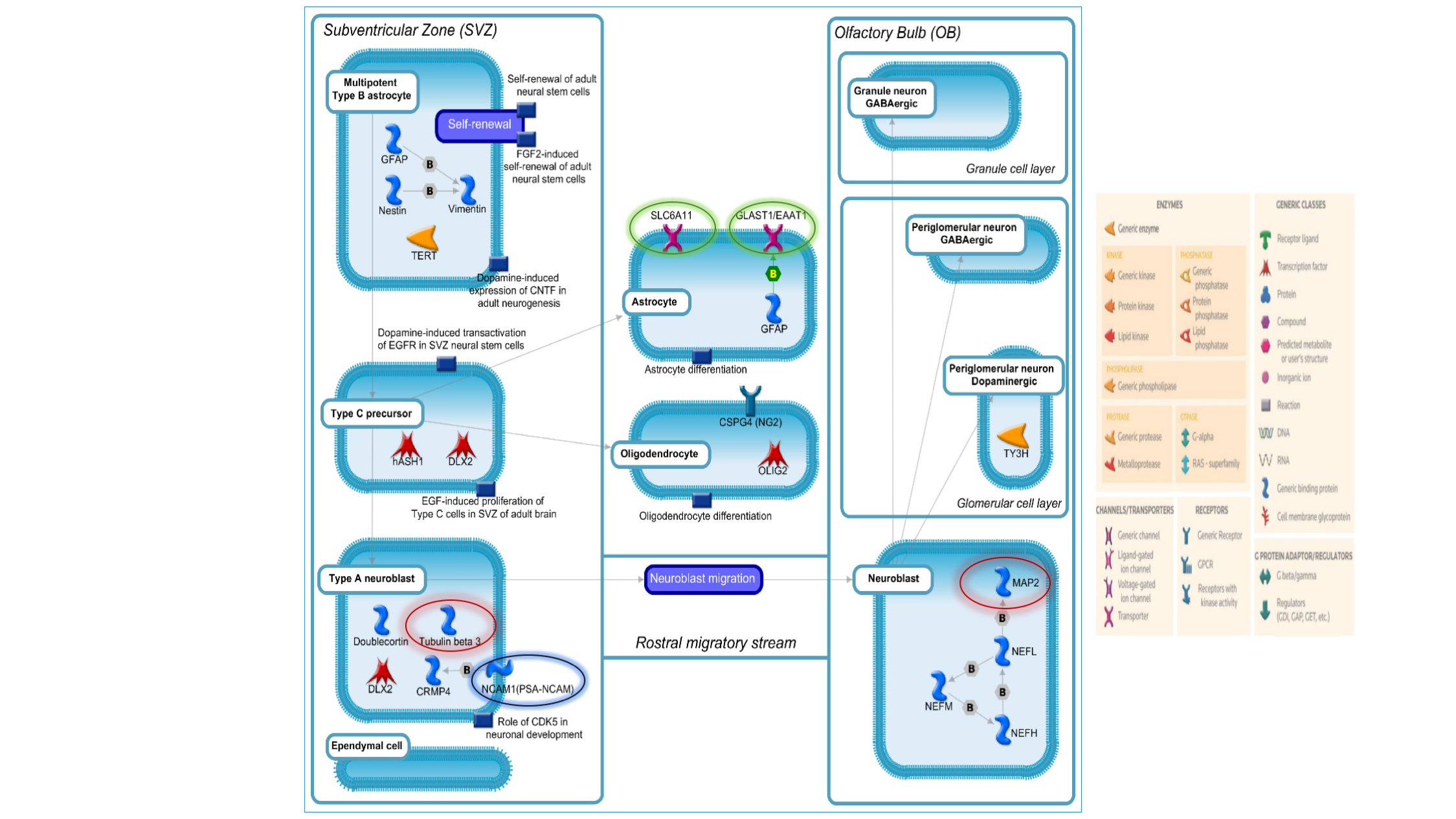

Supplement: Supplementary file 3 — Supplementary Figure S2. [file 41598_2020_70383_MOESM3_ESM.tiff]

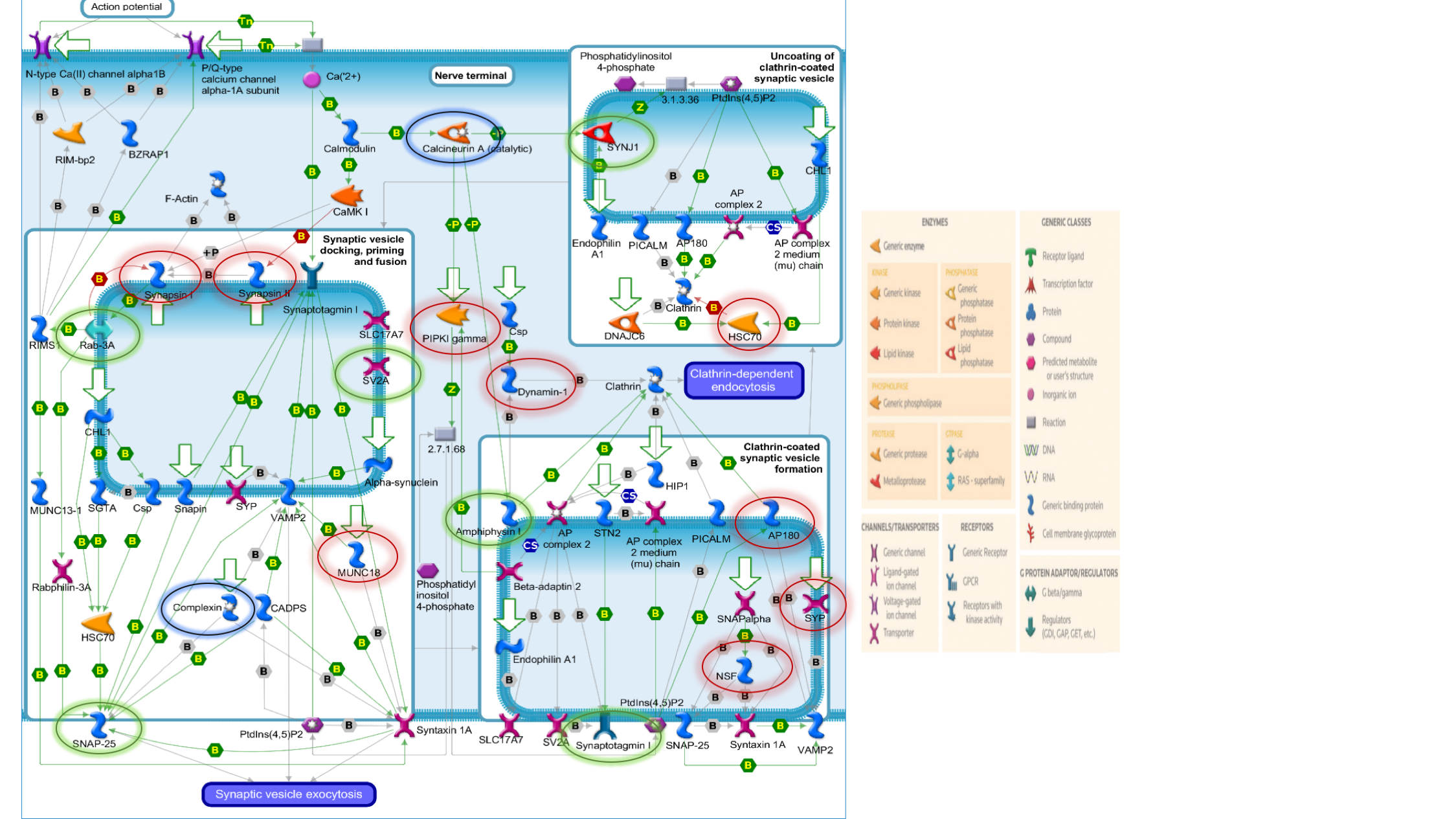

Supplement: Supplementary file 4 — Supplementary Figure S3. [file 41598_2020_70383_MOESM4_ESM.tiff]

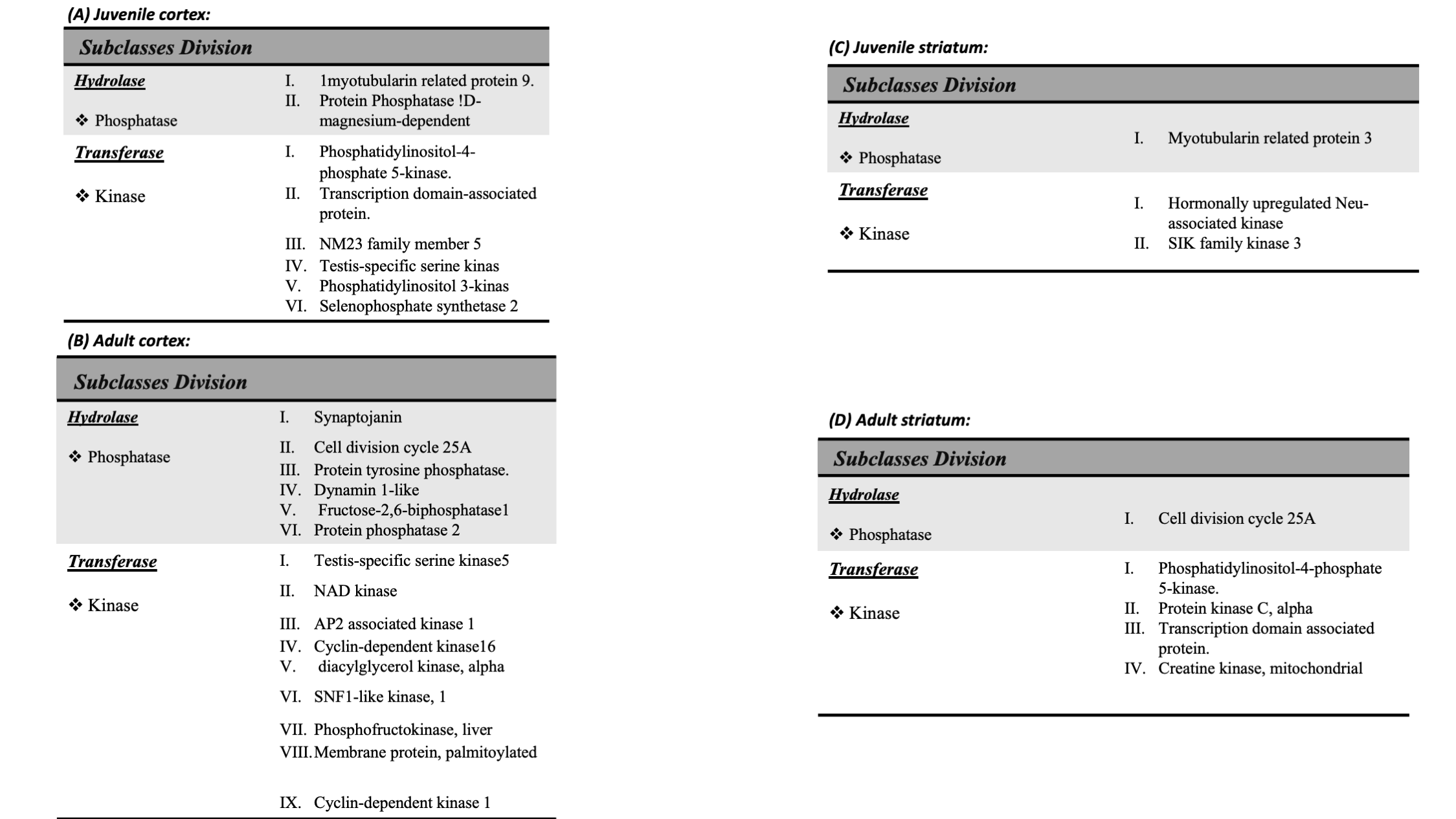

Supplement: Supplementary file 5 — Supplementary Figure S4. [file 41598_2020_70383_MOESM5_ESM.tiff]

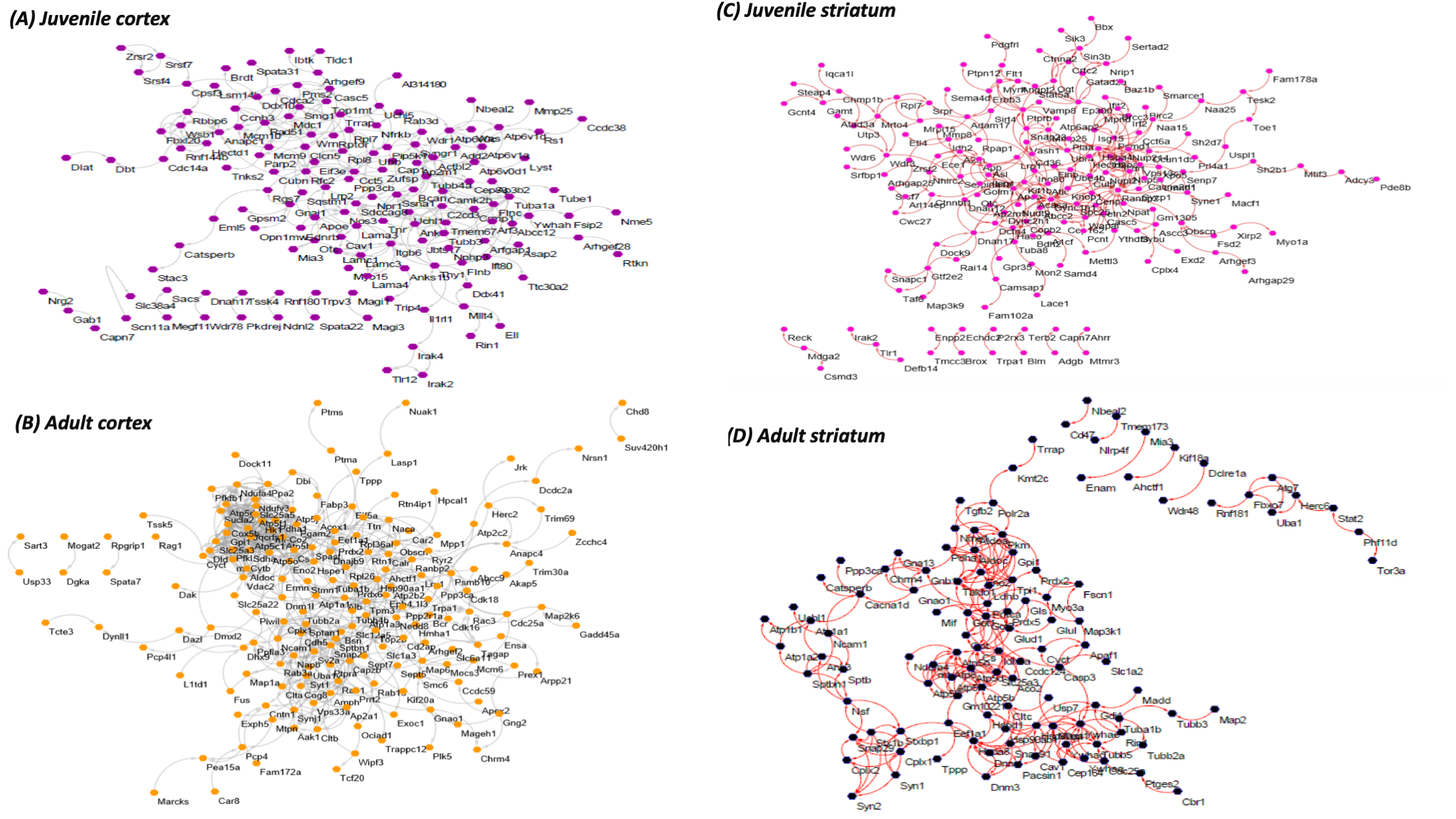

Supplement: Supplementary file 6 — Supplementary Figure S5. [file 41598_2020_70383_MOESM6_ESM.tiff]

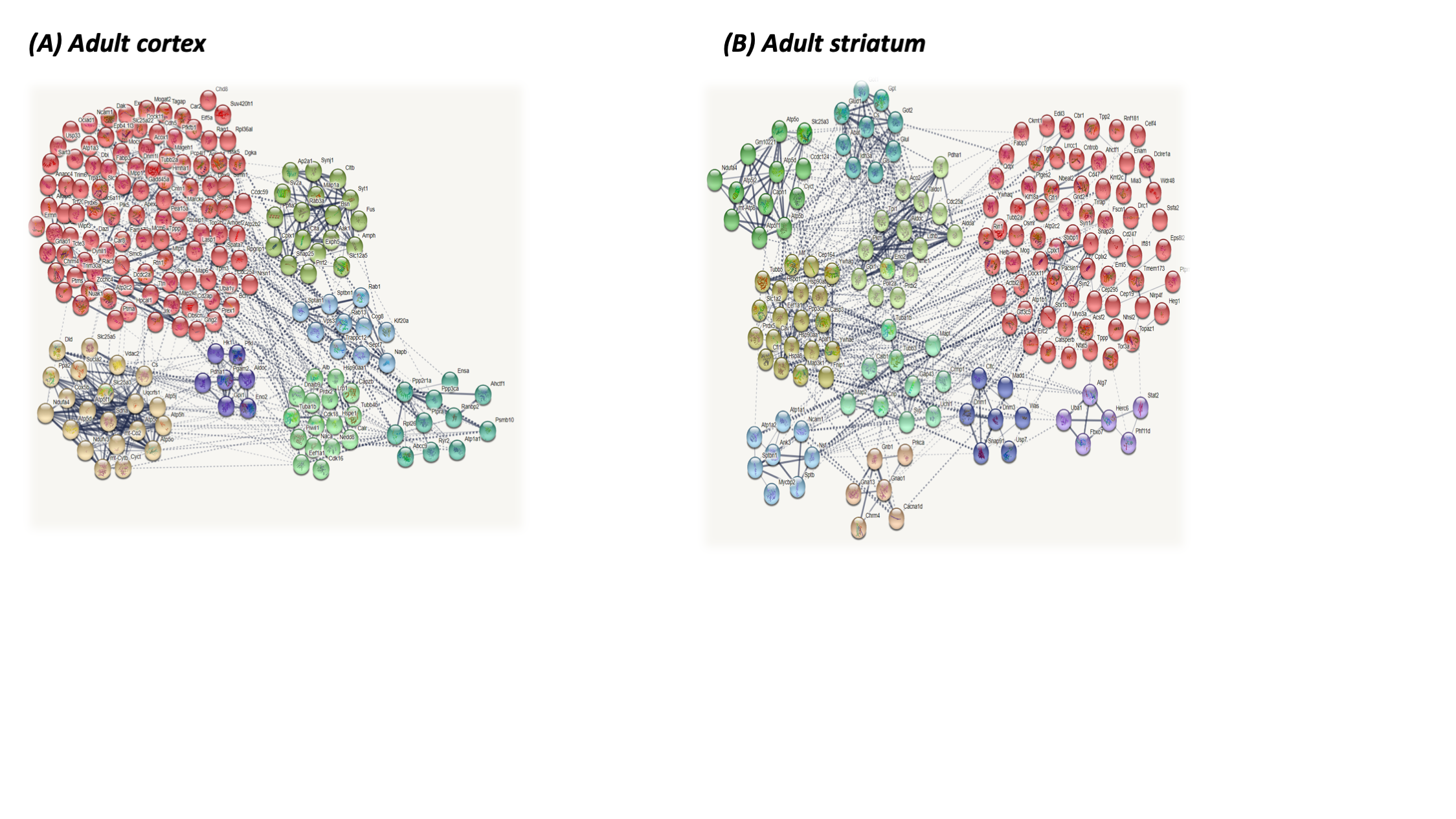

Supplement: Supplementary file 7 — Supplementary Figure S6. [file 41598_2020_70383_MOESM7_ESM.tiff]
